# Single‐Pocket‐Incision Generator Exchange From a Subcutaneous to an Extravascular Implantable Cardioverter‐Defibrillator

**DOI:** 10.1002/joa3.70391

**Published:** 2026-06-11

**Authors:** Yoshimi Onishi, Saori Chino, Masaaki Kurata, Yoshitaka Iso, Hiroshi Suzuki

**Affiliations:** ^1^ Division of Cardiology, Department of Medicine Showa Medical University Tokyo Japan; ^2^ Division of Cardiology, Department of Medicine Showa Medical University Fujigaoka Hospital Yokohama Kanagawa Japan

**Keywords:** Brugada syndrome, extravascular implantable cardioverter‐defibrillator, inappropriate shock, single‐incision strategy, subcutaneous implantable cardioverter‐defibrillator

## Abstract

Subcutaneous‐to‐extravascular implantable cardioverter‐defibrillator exchange can create a practical challenge: the existing subcutaneous generator pocket lies posteriorly, whereas the new extravascular generator site is planned more anteriorly. In this case, fluoroscopic planning showed adjacent generator sites, allowing a single intervening pocket incision. Bidirectional dissection enabled subcutaneous generator removal and extravascular pocket creation, while separate deep‐layer closure avoided a large continuous pocket and preserved standard extravascular generator positioning.

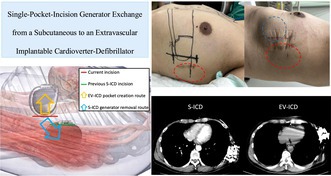

The extravascular implantable cardioverter‐defibrillator (EV‐ICD) has demonstrated durable safety and performance as a nontransvenous defibrillation system, with low inappropriate shock rates [1]. Conversion from a subcutaneous implantable cardioverter‐defibrillator (S‐ICD) to an EV‐ICD has been reported in patients with Brugada syndrome and recurrent inappropriate S‐ICD shocks due to oversensing [2]. However, the incision strategy for same‐session conversion remains poorly defined. Because the S‐ICD generator is usually located in a posterior pocket and the EV‐ICD generator is positioned more anteriorly, conversion may intuitively be approached through 2 separate incisions. We describe a single‐pocket‐incision generator exchange strategy planned according to the relative positions of the existing S‐ICD pocket and the fluoroscopically defined EV‐ICD generator site.

A 55‐year‐old man with Brugada syndrome (height, 171 cm; weight, 67 kg; body mass index, 22.9 kg/m^2^) underwent S‐ICD implantation after ventricular fibrillation and received 2 appropriate shocks during 7 years of follow‐up. One year before conversion, he experienced both an appropriate shock and an inappropriate shock due to T‐wave oversensing with double counting. Epicardial substrate ablation performed 6 months before conversion eliminated the Brugada electrocardiographic pattern (Figure 1). Postablation S‐ICD sensing‐vector assessment, performed immediately after ablation and again after recurrent inappropriate shocks, showed consistent results: the primary and alternate vectors passed screening, whereas the secondary vector failed because of low R‐wave amplitude. Nevertheless, 2 inappropriate shocks due to T‐wave oversensing occurred during use of the alternate vector with the Smart Pass filter enabled. After reprogramming to the primary vector, in which the Smart Pass filter could not be enabled because of low R‐wave amplitude, 2 additional inappropriate shocks due to T‐wave oversensing occurred. EV‐ICD conversion was therefore planned to prevent further inappropriate S‐ICD therapy while preserving a nontransvenous defibrillation strategy.

**FIGURE 1:**
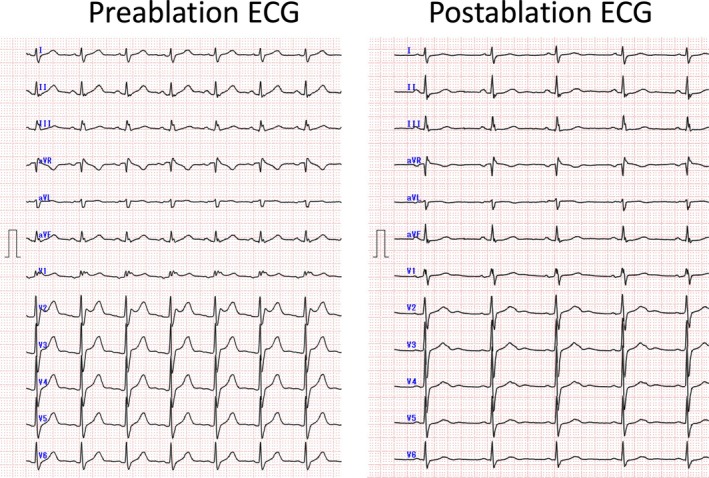
Twelve‐lead electrocardiograms before and after epicardial substrate ablation, showing elimination of the Brugada electrocardiographic pattern.

The EV‐ICD generator site was defined using the recommended lateral fluoroscopic approach before skin incision. The planned EV‐ICD generator position was located immediately anterior to the existing S‐ICD generator site, making a single‐incision approach feasible. Preoperative skin marking incorporated the existing S‐ICD pocket, the fluoroscopically defined EV‐ICD generator site, and the intervening skin incision. Instead of placing the incision directly over either generator site, we placed it between the 2 pockets to allow dissection toward both pockets through the same incision. The postoperative wound remained limited to this single intervening incision, while the implanted EV‐ICD pocket and previous S‐ICD pocket were separately identifiable on skin marking (Figure 2).

**FIGURE 2:**
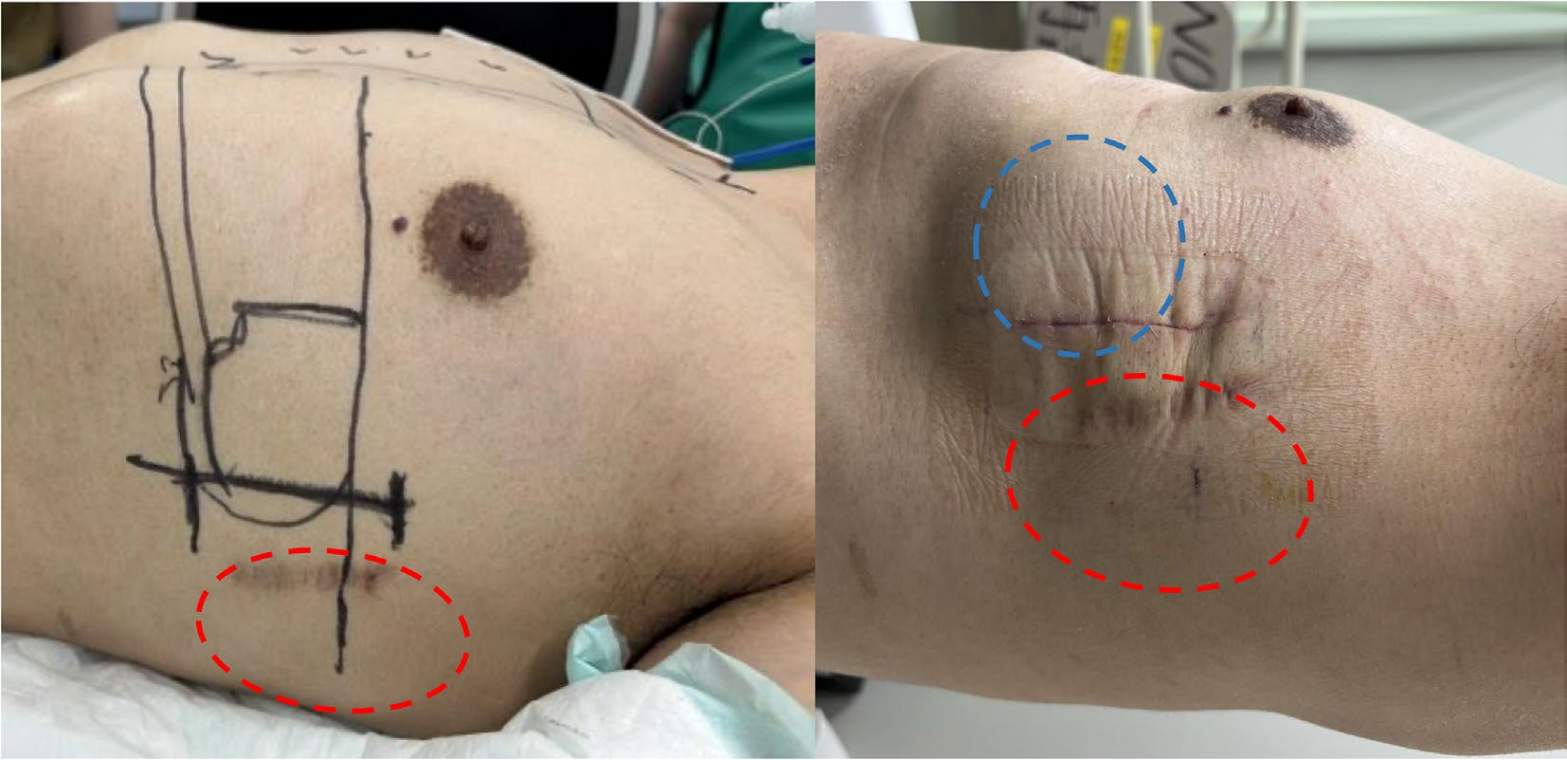
Preoperative marking and postoperative wound appearance showing the intervening single skin incision between the previous subcutaneous implantable cardioverter‐defibrillator pocket and the newly created extravascular implantable cardioverter‐defibrillator pocket.

Through the same intervening incision, the S‐ICD generator was removed by dissecting inferiorly and posteriorly toward the preexisting S‐ICD pocket. The EV‐ICD pocket was then created through the same skin incision by dissecting in the opposite direction, superiorly and anteriorly, toward the fluoroscopically planned generator site. Thus, EV‐ICD pocket creation was performed from an inferior incision toward a superior‐anterior generator position, effectively reversing the usual direction of pocket dissection. Because the final EV‐ICD generator position was superior and anterior to the incision, its suture‐anchoring site lay in the deeper superior portion of the pocket. Despite this greater depth, secure fixation was achieved without adding a second incision. Although both procedures were performed through a single skin incision, the deeper tissue layers between the S‐ICD explantation cavity and the newly created EV‐ICD pocket were closed separately at the muscle‐layer level to avoid creating one large continuous pocket between the 2 generator sites. Defibrillation testing was successful at 30 J, with a shock impedance of 37 Ω, and the shock impedance remained satisfactory at 53 Ω on the following day. Paired pre‐ and postconversion computed tomography confirmed that the final EV‐ICD generator was positioned immediately anterior to, and closely aligned with, the original S‐ICD generator site (Figure 3). During 3 months of follow‐up after EV‐ICD implantation, no shocks, antitachycardia pacing therapies, or oversensing episodes were recorded by remote monitoring or in‐office device interrogation.

**FIGURE 3:**
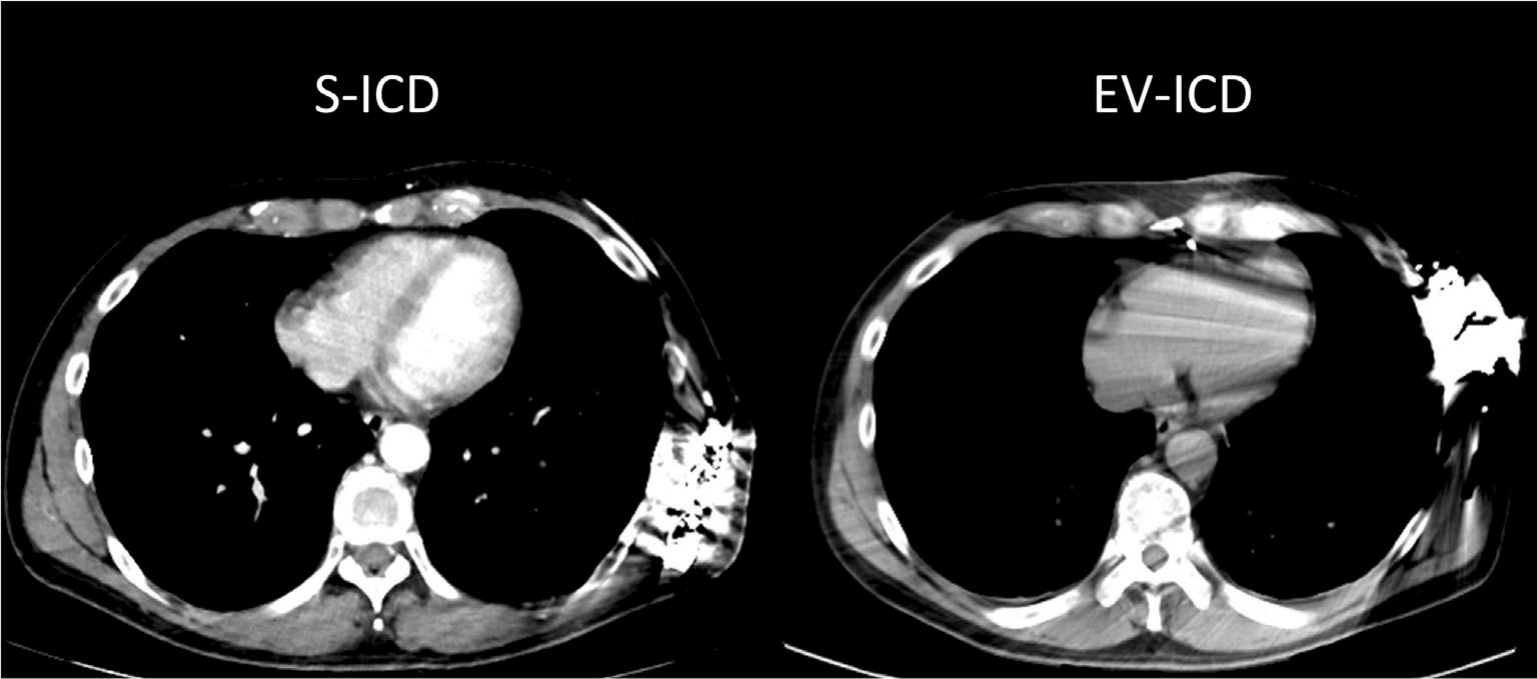
Pre‐ and postconversion computed tomography demonstrating that the final extravascular implantable cardioverter‐defibrillator generator position was immediately anterior to and closely aligned with the original subcutaneous implantable cardioverter‐defibrillator generator site.

This case illustrates an incision strategy for same‐session S‐ICD to EV‐ICD generator exchange based on the actual relationship between the old and new generator sites. The S‐ICD generator is typically positioned posteriorly between the serratus anterior and latissimus dorsi muscles, whereas the EV‐ICD generator is positioned more anteriorly using lateral fluoroscopic guidance. Thus, S‐ICD explantation and EV‐ICD implantation may appear to require separate operative fields and separate skin incisions. In practice, however, whether a single incision can be used depends on how closely the preexisting S‐ICD pocket lies to the planned EV‐ICD generator site.

In the present patient, the 2 generator sites were close enough to allow an intervening incision that provided inferior‐posterior access for S‐ICD explantation and superior‐anterior access for EV‐ICD pocket creation (Figure 4). This strategy may not be limited to markedly lean patients, provided that the 2 generator sites are adjacent or partially overlapping. Conversely, in patients with a larger body habitus or widely separated generator sites, a single‐incision approach may be unsuitable or may risk compromising optimal EV‐ICD generator positioning. Thus, preoperative assessment of the relationship between the existing S‐ICD pocket and the fluoroscopically planned EV‐ICD site is essential.

**FIGURE 4:**
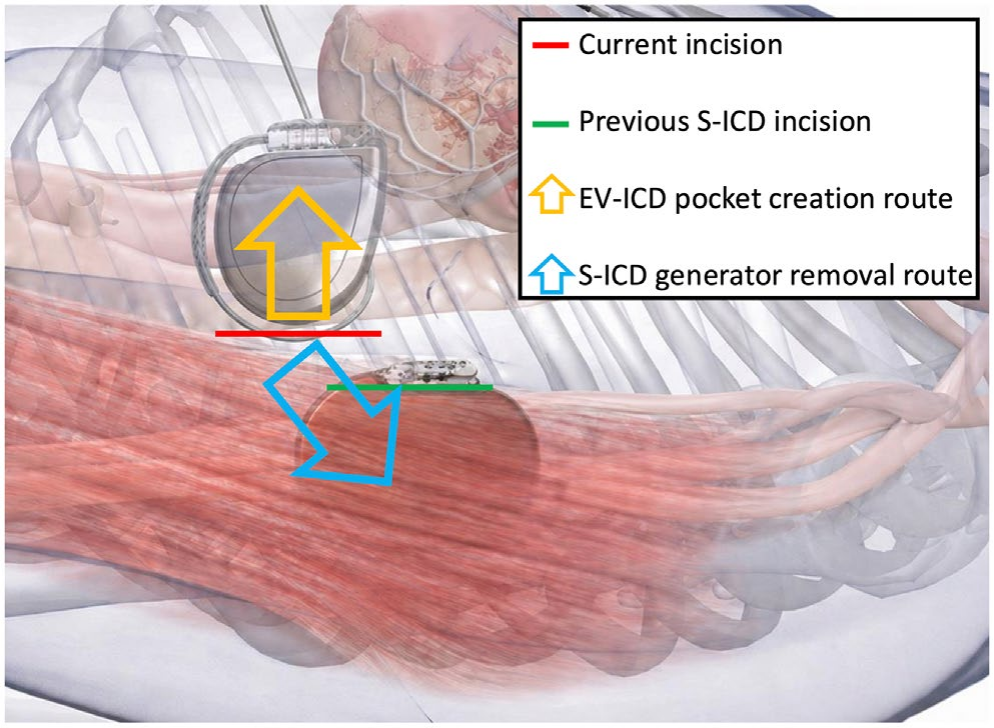
Schematic illustration of bidirectional access through the intervening single incision, with inferior‐posterior dissection for subcutaneous implantable cardioverter‐defibrillator removal and superior‐anterior dissection for extravascular implantable cardioverter‐defibrillator pocket creation.

An important technical point is to avoid leaving the S‐ICD explantation cavity and the new EV‐ICD pocket as one large continuous deep pocket. Such an oversized generator space could lower shock impedance and, if anchor fixation failed, permit substantial generator displacement. Therefore, although both procedures were performed through a single skin incision, the deeper tissue layers between the 2 generator sites should be closed separately to maintain pocket stability. Same‐session conversion may also avoid separate hospitalizations, repeated anesthesia, and an interval without implanted defibrillator protection. In anatomically suitable patients, this single‐incision strategy may reduce wound burden, postoperative pain, the need for wound care at multiple sites, and cosmetic concerns without compromising standard EV‐ICD generator positioning.

## Funding

The authors have nothing to report.

## Ethics Statement

The authors have nothing to report.

## Consent

The patient has provided consent for publication.

## Conflicts of Interest

The authors declare no conflicts of interest.

## Data Availability

The data supporting the findings of this study are available from the corresponding author upon reasonable request.
